# Interpersonal trauma and its relation to childhood psychopathic traits: what does ADHD and ODD add to the equation?

**DOI:** 10.1186/s12888-021-03610-7

**Published:** 2021-12-18

**Authors:** John J. Marshall, Karolina Sörman, Natalie Durbeej, L. Thompson, Sebastian Lundström, Helen Minnis, Clara Hellner, Christopher Gillberg

**Affiliations:** 1 Institute of Health and WellbeingUniversity of Glasgow, Glasgow, UK; 2 Department of Clinical Neuroscience, Karolinska Institutet, Stockholm, Sweden; 3 Department of Public Health and Caring Sciences, Uppsala University, Stockholm, Sweden; 4 Gillberg Neuropsychiatry Centre, Sahlgrenska Academy, Gothenburg University, Glasgow, UK; 5 Institute of Neuroscience and Physiology, Gillberg Neuropsychiatry Centre, Centre of Ethics Law and Mental Health, Gothenburg University, Gothenburg, Sweden

**Keywords:** Child, Psychopathy, Trauma, ADHD, ODD, Neurodevelopmental disorders

## Abstract

**Background:**

Childhood trauma has demonstrated associations with callous-unemotional traits (e.g., reflecting lack of remorse and guilt, unconcern about own performance). Less is known about associations between trauma and multiple domains of child psychopathic traits. There has also been limited focus on the role of co-occurring disorders to psychopathy traits among children, namely, attention-deficit hyperactivity disorder (ADHD) and oppositional defiant disorder (ODD) and how they interact with childhood trauma.

**Methods:**

We examined to what degree childhood interpersonal trauma can predict parent-rated psychopathic traits in a large population based Swedish twin sample (*N* = 5057), using a stringent definition of interpersonal trauma occurring before age 10. Two hundred and fifty-one participants met the interpersonal trauma criteria for analysis. The study explored the additional impact of traits of attention-deficit hyperactivity disorder (ADHD) and oppositional defiant disorder (ODD).

**Results:**

Linear regressions demonstrated statistically significant but clinically negligible effects of interpersonal trauma on total and subscale scores of parent-rated psychopathic traits. When exploring interaction effects of ADHD and ODD into the model, the effect increased. There were interaction effects between ODD and trauma in relation to psychopathic traits, suggesting a moderating role of ODD. Having been exposed to trauma before age 10 was significantly associated with higher parent rated psychopathy traits as measured by The Child Problematic Traits Inventory-Short Version (CPTI-SV), however the explained variance was small (0.3–0.9%).

**Conclusions:**

The results challenge the notion of association between interpersonal trauma and youth psychopathic traits. They also highlight the need to gain an improved understanding of overlap between psychopathic traits, ADHD and ODD for clinical screening purposes and the underlying developmental mechanisms.

## Background

Research on youth psychopathic traits has mainly focused on callous unemotional (CU) traits reflecting a lack of remorse and guilt and unconcern about own performance [1]. Factor analytic studies, however, have demonstrated the multi-dimensional nature of psychopathic traits in youth across measures [2]. However, youth psychopathic traits are proposed to encompass several domains, including grandiose-manipulative (GM), and daring-impulsive (DI) traits [3]. Antisocial outcomes are more likely to be predicted by the combination of the three domains of traits, rather than CU traits alone [4, 5]. Adult antisocial outcomes are more likely to be predicted by the combination of childhood interpersonal, affective, and behavioral traits than CU traits alone [4, 5]. For example, interpersonal psychopathic traits may be uniquely related to bullying, relational aggression, and delinquency, more so than CU traits [6]. Moreover, studies concerned with cognitive functioning have found that GM traits within the psychopathy construct are critical to unprovoked aggression [7]. Childhood studies are mainly concerned with CU traits and have not fully captured the construct of psychopathy, thereby potentially limiting risk prevention efforts. To understand psychopathic traits as a broad neurodevelopmental condition rooted in early childhood, it is important to investigate the manifestation and correlation of all three components of psychopathic traits (i.e., also including impulsive and grandiose traits).

Childhood psychopathic traits manifesting in early or middle childhood are an important (albeit moderate) predictor of adolescent delinquency, general recidivism, and violent recidivism [8]. Such traits also constitute a critical risk factor for adult psychopathy, antisocial behavior and substance abuse [1].

### Trauma and psychopathic traits

Childhood trauma or adversity can be among several factors predicting adult psychopathy [9]. Even though research on childhood psychopathic traits has mainly focused on heritable pathways, risk trajectories, and neurobiological substrates [10], some research has explored associations between trauma and psychopathic traits. For example, a systematic review demonstrated how parenting styles may modulate the expression of CU traits [11]. Risk factors in the parental style that may exacerbate CU traits include reduced quality of parent–child affective interactions, reduced eye contact, lower observed dyadic warmth and lower parental sensitivity [12]. Harsh parenting experienced in early childhood [13, 14], corporal punishment [15], psychological aggression, non-consistent discipline experienced in middle childhood [16], and poor parent–child communication during adolescence [17] all predict higher levels of CU traits. Conversely, positive aspects of parenting may assist in the prevention of CU traits. In summary, problematic parenting may intensify CU traits, suggesting that childhood interpersonal trauma (CIT) may also have an amplifying effect on psychopathic traits. There are no studies concerning childhood interpersonal trauma and the full range of psychopathic traits.

### Childhood interpersonal trauma and psychopathic traits

CIT (e.g., physical, emotional, sexual abuse, physical and emotional neglect) is associated with a broad range of mental disorders in childhood and adulthood, including personality disorders (Waxman et al., 2014) [18–21]. Adult offenders consistently report higher levels of CIT compared to normative samples (Waxman et al., 2014) [21], and meta-analytic data has demonstrated a strong relationship between adverse childhood experiences (ACES), including CIT-perpetrating adult interpersonal violence and a range of mental disorders [22].

CIT has been proposed as a critical factor in the development of psychopathic traits; however, most studies have focused on the association between previous trauma and psychopathy in adulthood, asking participants to recall any previous CIT. In some retrospective studies, psychopathic adults have reported extensive CIT where the strongest associations are with physical abuse [23]. In other forms of CIT (e.g., neglect), blunted affective expression, alexithymia and the unemotional features of psychopathy have been identified as concomitants, implying a direct link between aspects of psychopathy and CIT [24]. Some studies indicate that putative CIT environmental factors such as parental rejection, neglect, and abuse are critical in the etiology of antisocial behavior and adult psychopathy [25–27]. One study demonstrated that adults with psychopathy who had committed murder, rape, or child abuse were relatively more likely to report childhood interpersonal trauma than those who were not psychopathic [28]. In particular, physical or emotional abuse and neglect have been associated with interpersonal callousness [29]. In addition, research has identified prospective links between general psychosocial adversity and CU traits, including high levels of chaos in the home [30].

### Psychopathic traits and neurodevelopmental disorders (NDDs)

Some youth with psychopathic traits present with a complex range of co-occurring neurodevelopmental disorders (NDDs). NDDs share symptomatology and etiology with each other and one diagnosis in childhood may be labeled as another disorder in adulthood [31]. For example, psychopathic traits, in particular impulsivity, tend to co-occur with symptoms of attention-deficit hyperactivity disorder (ADHD), reflecting poor concentration, impulsivity, and overactivity [32–34]. Psychopathic traits remain significantly higher in young offenders with ADHD after controlling for age, substance abuse, and early childhood adversities [35]. Conversely, previous studies have demonstrated that children with conduct problems and co-occurring psychopathic traits have higher levels of fearlessness and ADHD symptoms, compared to children with conduct problems and CU traits alone [36].

Research exploring the etiological underpinnings of co-occurring psychopathic traits and NDDs, particularly for interpersonal trauma, is notably lacking. Studies that are genetically sensitive, taking account of familial confounding on the effects of childhood traumatic experience on child and adult psychopathology, have revealed a shared liability in the causal role of traumatic events and NDDs [37]. Dinkler et al. [37] found that the co-occurrence of childhood maltreatment and NDD symptoms to a large extent was accounted for by a shared genetic liability, increasing both the risk of being maltreated and of having more co-occurring NDDs. Psychopathic traits in childhood could be at least partly transdiagnostic, interacting with the complexity of other neurodevelopmental comorbidities. Whether CIT is associated with the several dimensions of childhood psychopathic traits, taking account of NDDs, has not been established with a child population sample as opposed to stand-alone CU traits. The role of childhood interpersonal trauma factors impacting childhood psychopathy traits via NDDs such as ADHD is unknown, though such problems could feasibly exacerbate psychopathy via NDDs given how inattention and impulsivity might interact with core psychopathy traits.

This study examines to what degree childhood interpersonal trauma can predict parent-rated psychopathic traits in a large population-based Swedish twin sample. The study has two unique features: (*i*) a stringent definition of childhood interpersonal trauma occurring before age 10 and (*ii*) exploring the additional impact of traits of attention-deficit hyperactivity disorder (ADHD) and oppositional defiant disorder (ODD). This can further our knowledge of the pathways involved in the development of child psychopathic traits and may help inform the types of preventive interventions that are appropriate for this group of children.

## Methods

### Participants

In this study, data from the Child and Adolescent Twin Study in Sweden (CATSS) was used (see Anckarsäter et al. 2011 [38] for a description of the CATSS-study). Briefly, parents of all twins born in Sweden from the 1st of July 1992 are contacted in connection with the twins’ 9th birthday (the first 3 years of the study also included 12-year old’s) and asked to participate in a telephone interview (CATSS-9). At age 15, the twins and their parents are contacted again (CATSS-15) and asked to log on to a web page and fill out questionnaires. The CATSS-15 includes individuals born 1st January 1994 and onwards. Among participants in CATSS-9, 50% also responded in CATSS-15. In this study, participants born from January 1st, 1994, to June 31st, 1998, were included (as one of the instruments used was excluded from the study protocol in 1998). At the assessment at age 15, 5075 individuals had complete responses to A-TAC, CPTI-SV, and trauma. Thus, the final sample comprised 5057 participants among which 2408 (49.4%) were boys and 2463 (50.6%) were girls.

### Measures

**The Child Problematic Traits Inventory-Short Version (CPTI-SV)** was used to assess parent-rated psychopathic traits in CATSS-9. This is an abbreviated 12-item version of the 28-item Child Problematic Traits Inventory [39]. The CPTI was developed to assess all three dimensions of psychopathic traits in children, with three subscales: grandiose deceitful (GD), callous unemotional (CU), and impulsive need for stimulation (INS). The development of CPTI was theoretically based and guided by some prerequisites: included items have empirical support for being assessable in children and do not directly reflect rule breaking (e.g., conduct symptoms, antisocial behavior). CPTI has demonstrated adequate psychometric properties across studies, including excellent internal consistency and support for the three-factor structure using different informants (i.e., teachers and parents), genders and age groups [39]. Items of the CPTI-SV were selected by the test developer Andershed, based on the larger pool of CPTI-items. The selection was based on the perceived degree of correspondence with the three domains of psychopathic traits in the three-factor model of psychopathy (Andershed, personal communication) [6]. The CPTI-SV encompasses four items from the GD-subscale (e.g., “*Is often superior and arrogant towards others*”), four items from the CU-subscale (e.g., “*Usually does not seem to share others’ joy and sorrow*”), and four items from the INS-subscale (e.g., “*Often does things without thinking ahead*”). In line with the original scale, items were rated based on the child’s typical behavior with the following 4-point Likert scale: 1 (*does not apply at all*), 2 (*does not apply well*), 3 (*applies fairly well*), and 4 (*applies very well*). Alpha values were as follows: 0.79 (CPTI-total); 0.70 (GD); 0.77 (CU) and 0.67 (INS).

**The Life Stressor Checklist-Revised (LSC-R)** [40] was used to assess CIT. The LSC-R is a 30-item screening measure of traumatic life events according to DSM-IV criteria for posttraumatic stress disorder and other seriously stressful life events. The LSC-R has demonstrated adequate psychometric properties across studies, including good internal consistency [41]. In this study, CIT refers to interpersonal victimization, the elements of malevolence, betrayal, injustice, and immorality are more likely to be factors than in accidents, diseases, and natural disasters [38]. This definition of CIT did not include, for example, children, who were “victims” of accidents. To assess CIT at age 15, we used 9 dichotomous (i.e., yes/no) parent-reported questions (covering the following themes; emotional abuse; physical neglect; physical abuse; sexual touch; forced sex; witnessing physical violence in family; witnessing a threatening or violent incident; victim of hate crime; see appendix 1a for the questions). An open question was also asked, “Has the child ever been direct witness (not film, internet or TV) to any other serious incident that you have not mentioned?” Two authors (JM & KS) reviewed responses to this open question. A response that indicated an act directed towards the child or if the child was a direct witness was deemed as likely to have caused a traumatic response (e.g., seeing the mother being beaten, being exposed to mental abuse). Of the 419 answers on the open question, four were considered to reflect interpersonal trauma. If a parent endorsed a question about maltreatment, a follow-up question inquiring at what age the maltreatment had happened the first and/or the last time was asked. Only maltreatment reported to have occurred before the twins’ 10th birthday was included. In a subsequent step, participants who had not already reported trauma before age 10 but endorsed trauma at age 9 were added to the analysis. This assessment was based on three parameters: witnessing violence, being robbed, and trauma (including bullying, sexual abuse, and maltreatment, gauged through an open question). The responses to the open questions were reviewed by the two authors (JM & KS).

**The Autism-Tics, ADHD and other Comorbidities Inventory (A-TAC)** was used to assess symptoms of ADHD and ODD in CATSS-9. This measure has been validated in cross-sectional and longitudinal studies [42]. The A-TAC is a fully structured parental telephone interview measuring various domains of child and adolescent psychiatry. It was designed for use by laypeople over the phone. It consists of 96 symptom questions, which are asked from a lifetime perspective (Larson et al., 2014). The A-TAC measures ADHD and ODD domain symptoms.. All items in the A-TAC are coded ‘no’ (0), ‘yes, to some extent’ (0.5) and ‘yes’ [1]. The ADHD domain displays an area under the curve of .93 and α of .92 in a population-based sample including individuals who were diagnosed with ADHD before the age of 10 [42]. In the current study, the lower screening cut-off for ADHD symptoms (≥ 6) was used, with a sensitivity of .79 and a specificity of .90 [43]. ODD has been reported to have an AUC of .99 with α of .75. The cut-off for ODD was ≥3 with corresponding sensitivity and specificity of .88 and .97.

### Statistical analyses

Descriptive statistics (means, standard deviations, frequencies and percentages) were used to describe the sample and paired sample *t*-tests were used to compare means. Chronbach alpha values were calculated for assessment of internal consistency of the CPTI-SV instrument. Relations between trauma (a binary variable; yes/no) and psychopathic traits were explored using single and multiple linear regressions. In the multiple linear regression analysis, we investigated the single contributions of and interactions between trauma, ADHD, and ODD. The CPTI-SV total and subscale scores were used as dependent variables. We applied Bonferroni correction for multiple testing (=0.05/8) and thus *P*-values < 0.006 were considered statistically significant. All analyses were computed using SPSS, version 23, and STATA, version 14.

## Results

### Descriptives

At age 15, a total of 193 individuals endorsed having been exposed to our stringent definition of CIT before age 10. At age 9, the number of individuals reporting at least one type of interpersonal trauma (not already included at age 15) was 58. In total, therefore, 251 participants had been exposed to CIT before age 10.

A total of 306 participants (6.1%) reported symptoms of ADHD and 54 (1.1%) reported symptoms of ODD (see Table 1). Mean scores of the CPTI-SV scales in the sample and subgroups are demonstrated in Table 1.

**Table 1:** CPTI-SV scores (mean values) in the total sample, and subgroups with and without trauma before age 10

	CPTI-SV total ***M*** (***SD***)	GD ***M*** (***SD***)	CU ***M*** (***SD***)	INS ***M*** (***SD***)
*N* = 5057	1.36 (.30)	1.13 (.28)	1.14 (.31)	1.82 (.57)
**Subsample without trauma before age 10 (*n*** **= 4807)**	1.36 (.30)	1.12 (.27)	1.14 (.30)	1.81 (.56)
**Subsample with trauma before age 10 (*n*** **= 250)**	1.49 (.40)	1.21 (.40)	1.22 (.43)	2.02 (.66)

CPTI-SV Child Problematic Traits Inventory, *GD* Grandiose Deceitful, *CU* Callous Unemotional

*INS* Impulsivity Need for stimulation

### Relations between trauma, psychopathic traits and NDDs

Single linear regressions exploring associations between trauma and CPTI-SV scores are demonstrated in Table 2. Having been exposed to trauma before age 10 was significantly associated with higher CPTI-SV (across all scales); however, the explained variance was small (0.3–0.9%).

**Table 2:** Single linear regressions exploring the relations between trauma before age 10 (independent variable) and CPTI-SV total and subscale scores (dependent variables) (*n* = 5057)

	Standardized β (95% CI)	***P***	R^**2**^
Trauma before age 10			
CPTI-SV GD	.073 (.059–.129)	<.001	.005
CPTI-SV CU	.058 (.043.121)	<.001	.003
CPTI-SV INS	.082 (.144–.288)	<.001	.007
CPTI-SV Total	.095 (.095–.171)	<.001	.009

*CPTI-SV* Child Problematic Traits Inventory, *GD* Grandiose Deceitful, *CU* Callous Unemotional, *INS* Impulsivity Need for stimulation

Multiple linear regressions were computed to explore the impact of ADHD and ODD (Tables 3, 4, 5 and 6). As demonstrated, CPTI-SV total scores were predicted by trauma before age 10 and ADHD. The interaction between interpersonal trauma and ADHD was not significant (Table 3). Moreover, both trauma and ODD predicted higher CPTI-SV total scores, but there was no significant interaction between trauma and ODD. Both trauma and ADHD predicted higher CPTI-SV GD scores; however, there was no significant interaction between the two variables (Table 4). A corresponding pattern emerged when ODD was added to the model.

**Table 3:** Prediction of CPTI-SV total scores by multiple linear regressions with impact of trauma, ADHD and ODD, as well as interactions between trauma, ADHD or ODD. Standardized beta coefficients (β), *p-*values, 95% confidence intervals (CI), and R^2^ values are shown (*n* = 5057)

**Model**	**Independent variables**	**Standardized β**	***P***	**95% CI Lower**	**95% CI Upper**	**R**^**2**^
1	Trauma before age 10	.073	**<.001**	.035	.112	.143
	ADHD > cut-off	.430	**<.001**	.397	.463	
	Trauma before age 10 **x** ADHD > cut-off	.086	.087	−.012	.185	
2	Trauma before age 10	.097	**<.001**	.059	.135	.075
	ODD > cut-off	.644	**<.001**	.566	.722	
	Trauma before age 10 **x** ODD > cut-off	.186	.044	.005	.367	
**Model**	**Independent variables**	**Standardized β**	***P***	**95% CI Lower**	**95% CI Upper**	**R**^**2**^
1	Trauma before age 10	.073	<.001	.035	.112	.143
	ADHD	.430	<.001	.397	.463	
	Trauma before age 10 **x** ADHD > cut-off low	.086	.087	−.012	.185	
2	Trauma before age 10	.097	<.001	.059	.135	.075
	ODD > DSM cut-off	.644	<.001	.566	.722	
	Trauma before age 10 **x** ODD > DSM cut-off	.186	.044	.005	.367

**Table 4:** Prediction of **CPTI-SV GD scores** by multiple linear regressions with impact of trauma, ADHD and ODD, as well as interactions between trauma, ADHD or ODD. Standardized beta coefficients (β), *p-*values, 95% confidence intervals (CI), and R^2^ values are shown (*n* = 5057)

Model	Variables	Standardized β	***P***	95% CI Lower	95% CI Upper	R^**2**^
1	Trauma before age 10	.055	**.004**	.018	.093	.057
	ADHD > cut-off	.238	**<.001**	.207	.269	
	Trauma before age 10 **x** ADHD > cut-off	.082	.089	−.013	.177	
2	Trauma before age 10	.064	**<.001**	.029	.099	.075
	ODD > cut-off	.622	**<.001**	.550	.693	
	Trauma before age 10 **x** ODD > cut-off	.088	.298	−.077	.253

**Table 5:** Prediction of **CPTI-SV CU scores** by multiple linear regressions with impact of trauma, ADHD and ODD, as well as interactions between trauma, ADHD or ODD. Standardized beta coefficients (β), *p-*values, 95% confidence intervals (CI), and R^2^ values are shown (*n* = 5057)

Model	Variables	Standardized β	***P***	95% CI Lower	95% CI Upper	R2
1	Trauma before age 10	.051	.017	.009	.093	.045
	ADHD > cut-off	.242	**<.001**	.208	.277	
	Trauma before age 10 **x** ADHD > cut-off	.038	.482	−.068	.143	
2	Trauma before age 10	.046	**.002**	.007	.085	.043
	ODD > cut-off	.458	**<.001**	.377	.539	
	Trauma before age 10 **x** ODD > cut-off	.340	**<.001**	.154	.527

**Table 6:** Prediction of CPTI-SV INS scores by multiple linear regressions with impact of trauma, ADHD and ODD, as well as interactions between trauma, ADHD or ODD. Standardized beta coefficients (β), *p-*values, 95% confidence intervals (CI), and R^2^ values are shown (*n* = 5057)

Model	Variables	Standardized β	***P***	95% CI Lower	95% CI Upper	R^**2**^
1	Trauma before age 10	.111	**.003**	.038	.185	.139
	ADHD > cut-off	.805	**<.001**	.743	.866	
	Trauma before age 10 **x** ADHD > cut-off	.125	.187	−.061	.310	
2	Trauma before age 10	.176	**<.001**	.103	.248	.038
	ODD > cut-off	.859	**<.001**	.709	1.01	
	Trauma before age 10 **x** ODD > cut-off	.109	.536	−.237	.455	

When using CPTI-SV CU scores as an outcome, ADHD predicted higher scores, but trauma and the interaction term did not (Table 5). In contrast, both trauma and ODD predicted higher scores as well as the interaction between the two variables. Finally, trauma, ADHD, and ODD all predicted higher CPTI-SV INS scores but there was no interaction between trauma and ADHD or ODD.

## Discussion

This study set out to explore associations between childhood interpersonal trauma (CIT), all three domains of psychopathic traits, and NDDs including ADHD and ODD. The main finding is that CIT had a significant but negligible association with CPTI-SV-assessed youth psychopathic traits. The prediction of CU-traits traits by interpersonal trauma was small,, and moderated via ODD.

The absence of a childhood interpersonal trauma association with psychopathic traits in this study might indicate that maltreatment is a signal for the presence of neurodevelopmental disorders transmitted from adult to child via genetic factors. CU-traits are demonstrated to be moderately to strongly heritable [44]. Therefore, children’s pre-existing psychopathic traits may evoke negative parenting responses [45]. Conversely, differential susceptibility indicates that positive parenting may buffer the emergence of genetically underpinned psychopathy traits.

Studies concerned with maltreatment and childhood CU traits may highlight a complex reciprocal interplay between interpersonal trauma, genetic risk, and developmental status in emerging psychopathic traits. Maltreatment in the form of harsh parenting impacted younger children transitioning from toddlerhood to preschool more, albeit moderated by genetic risk for these traits [14]. It may be that there is a critical earlier developmental window for CIT to impact later emerging psychopathic traits and that more specific timing of CIT needs to be included in future research to better understand this. Alternatively, biological and underpinning temperamental drivers, such as fearlessness, for psychopathy traits may eclipse psychosocial factors, compared to a CIT-based etiology.

This finding runs counter to several previous studies on adults and youths, demonstrating some relationship between maltreatment (or problematic parenting) and psychopathic traits. It is important to point out, however, that previous studies have generated mixed findings. In a sample of youth (11–17 years old), Milone et al. (2019) demonstrated associations between maltreatment and externalizing behaviors, but not CU traits [46]. A recent systematic review found that primary psychopathy may precede the effects of adverse childhood experiences [47]. About 2% of our sample experienced CIT. In other population samples of similar-aged children, the incident rate of child abuse or maltreatment is reported at 2.5% [48].

The causal nature of CIT as an environmental factor impacting the propensity for psychopathic traits is difficult to determine without behavioral genetic methodologies such as co-twin designs to account for genetic and shared environmental confounding. Furthermore, there are parental effect causal assumptions underlying association studies [37]. Dinkler et al. [37] hypothesize that the parents’ genes related to their own neurodevelopmental traits are correlated with their children’s genetically influenced NDDs and at the same time may increase the risk of the parents maltreating their child. Moreover, Dinkler et al. found a small increase in symptoms of ADHD and Autism Spectrum Disorder in maltreated MZ twins as compared with their non-maltreated co-twins [37].

The results demonstrated a potential moderating role of ODD, in the associations between psychopathic traits, especially CU traits, and trauma. ODD is characterized by defiant, disobedient, and hostile behavior towards authority figures. Where children present with ODD, this may put greater strain on parents, adversely affect their sense of competence, and child ODD behaviors, in particular, may be more influential in engendering hostile negative parenting behaviors than vice versa [49]. Parents are known to use harsh parenting where children have callous-unemotional traits along with conduct problems or other externalising behavior problems, such as ODD, compared to CU traits alone [49]. Additional layers of oppositionality among children with psychopathic traits may evoke greater risk for parental maltreatment. Core deficits in children with psychopathic traits, such as their ability to recognize others’ distress cues [42] and pervasive lack of concern for others’ feelings [43], are likely to engender negative behaviors from peers and parents, particularly where the latter are also experiencing NDDs, compromising parenting capacity. We identified, however, a interaction effect for CIT and ODD for CU traits. We speculate that the presence of ODD and CU traits is a critical vector in disrupting attachments thereby increasing the likelihood of evoking CIT. CU traits are implicated in the abnormal development of conscience and morality. A fearless temperament is also linked to emerging externalising conduct problems. The attenuation of moral development processes along with increases in externalising conduct problems might arouse a greater chance of rupture to parent/carer attachment, compared to ODD and GU, or ODD and INS traits.

This study did not find an important relationship between CIT and psychopathy traits perhaps because of causal models involving a developmental cascade from genetic factors to early trait/temperament precursors and their interactions with parental factors across different developmental periods. Causal mechanisms can only be elucidated by genetically informed research designs.

Given the moderating role of ODD, it is important to study different combinations of neuro-developmental problems along with psychopathic traits. For example, the combination of ADHD symptoms may be linked to particularly high-risk pathways of callousness, impulsivity, or narcissism [50, 51]. ODD is also often co-morbid with ADHD and can be associated with poorer life outcomes. Such combinations of ODD, ADHD, and psychopathic traits may pose a greater parenting challenge than psychopathic traits alone and increase the likelihood of maltreatment, leading to CIT. The early identification of combinations of overlapping NDDs among children with psychopathic traits may, therefore, be critical for prevention. Treatment for co-occurring NDDs such as ODD and ADHD may be important in deflecting negative outcomes for children with psychopathic trait trajectories. Further behavioral genetic research using co-twin design methods is needed to disentangle etiological gene x environment factors with the inclusion of CIT.

### Strengths and limitations

This study has several strengths, including a prospective design, large sample size, assessment of all three dimensions of psychopathic traits, and the stringent definition of childhood interpersonal trauma. Moreover, the community sample used in the present study also should make the results more generalizable than association studies based on clinical, incarcerated, or offending samples. The study is also disadvantaged by several limitations. Children aged 9 were added to the analysis based on an open question reviewed by the authors in contrast to the children at 15 assessed by the LSC-R. However, careful review of open-ended question responses were assessed to ensure parity of interpersonal trauma definitions between the age 15 and age 9 groups. In contrast to the other scales used, the CPTI-SV has not been formally validated (i.e., no factor analysis has been conducted). Therefore, subscale scores should be interpreted with caution. The trauma variable was binary (yes/no) and provided no further information regarding severity of trauma (e.g., number of different traumas experienced, length of experience). It reflected CIT in a broader sense, there was no possibility to further specify subtype of CIT. CIT was rated by parents/carers. Where trauma resulted from parental abuse of neglect, parental evaluation may have led to underestimate of trauma. There are limitations with regard to the ODD scale with low sensitivity and specificity. Moreover, we did not include analyses on comorbid diagnoses in participants (e.g., comorbid ODD and ADHD). Given that previous literature has demonstrated that the prognosis of such comorbidity could be problematic compared to individuals with ADHD alone, this can be considered a limitation of the study. Future studies should apply a genetically informed research design to infer the causal mechanisms with regards to CIT in population cohorts. Studies should also apply the broad conceptualisation of psychopathic traits rather than CU traits alone and consider the differential impacts of primary versus secondary psychopathy in the context of CIT. Given that the LSC-R is parent reported in this study, biases might have been introduced (see Dinkler et al. [37] for a discussion of validity and reliability of the LSC-R in previous studies.)

## Conclusion

CIT confers negligible variance for psychopathy traits among children in this study, contrary to retrospective evidence among psychopathic adults and to studies of hostile parenting among younger children with CU traits who are at risk of developing psychopathy. The variance of psychopathy traits explained by CIT in the context of co-occurring ADHD and ODD remains low. The need for genetically informed studies that consider CIT at various stages of childhood risk for psychopathy traits is critical along with how psychopathy traits interact with cascade in combination with other disorders such as ADHD and ODD.

## Data Availability

Data is available upon reasonable request.

## Abbreviations

ADHD: Attention Deficit Hyperactivity Disorder
ODD: Oppositional Defiant Disorder
CU Traits: Callous Unemotional
NDD: Neurodevelopmental Disorder
GM: Grandiose Manipulative
GD: Grandiose Deceitful
INS: Impulsive need for stimulation
DI: Daring Impulsive
CIT: The Child Problematic Traits
CPTI: The Child Problematic Traits Inventory
CPTI-SV: The Child Problematic Traits Inventory-Short Version
CATSS: Child and Adolescent Twin Study in Sweden
A-TAC: The Autism-Tics, ADHD and other Comorbidities Inventory
LSC-R: The Life Stressor Checklist-Revised
